# Intraocular Pressure and Anterior Segment Morphometry Changes after Uneventful Phacoemulsification in Type 2 Diabetic and Nondiabetic Patients

**DOI:** 10.1155/2019/9390586

**Published:** 2019-06-10

**Authors:** João N. Beato, David Reis, João Esteves-Leandro, Manuel Falcão, Vítor Rosas, Ângela Carneiro, Fernando Falcão Reis

**Affiliations:** ^1^Department of Ophthalmology, Centro Hospitalar Universitário de São João, Porto, Portugal; ^2^Department of Surgery and Physiology, Faculty of Medicine, University of Porto, Porto, Portugal; ^3^Faculty of Medicine, University of Porto, Porto, Portugal

## Abstract

**Purpose:**

To compare intraocular pressure (IOP) and anterior segment (AS) morphometry changes after uneventful phacoemulsification between nonglaucomatous eyes with open-angles from patients with and without type 2 diabetes mellitus (DM) and determine which factors may predict greater IOP-lowering effect.

**Methods:**

Forty-five diabetic (45 eyes) and 44 (44 eyes) age- and sex-matched non-DM patients with age-related cataract were enrolled in this prospective observational study. Goldmann applanation tonometry and AS Scheimpflug tomography (Pentacam® HR) were performed preoperatively and at 1- and 6-month follow-up. Linear regression analysis was performed to evaluate the clinical variables related to postoperative IOP changes at 6 months.

**Results:**

There was a significant postoperative IOP reduction 6 months after surgery (*p* < 0.001) by an average of 2.9 ± 2.9 mmHg (15.5%) and 2.4 ± 2.8 mmHg (13.0%) in the DM group and non-DM groups (*p* = 0.410), respectively. All AS parameters (anterior chamber depth, volume, and angle) increased significantly postoperatively (*p* < 0.001). Multivariate linear regression analysis showed that higher preoperative IOP was significantly associated with IOP reduction at 6-month follow-up (*p* < 0.05).

**Conclusion:**

Nonglaucomatous eyes with open-angles from both type 2 diabetic and nondiabetic patients experienced similar AS changes and IOP reductions following uneventful phacoemulsification, and this IOP-lowering effect was strongly correlated with preoperative IOP.

## 1. Introduction

Over the last two decades, several studies have consistently shown a significant and sustained intraocular pressure (IOP) decrease after uneventful phacoemulsification cataract surgery and posterior chamber intraocular lens (IOL) implantation in eyes either with or without ocular hypertension or glaucoma [1]. Although the pressure lowering mechanisms remain under debate, an improved aqueous access to the trabecular meshwork undoubtedly plays an important role, especially in eyes with partially or completely closed angles [2–5].

Anterior segment (AS) imaging has become progressively attractive with the advent of new high-resolution noncontact technologies, such as Scheimpflug-based systems (e.g., Pentacam® HR). These devices enable objective evaluation and quantification of several AS parameters (anterior chamber depth (ACD), volume (ACV), and angle (ACA)) [6], which have been studied as predictive markers of IOP reductions following cataract surgery [7–11].

The relationship between AS biometric changes and elevated plasma glucose concentrations in diabetes mellitus (DM) has been studied in the past. Most importantly, diabetic patients have been found to have thicker lenses and shallower anterior chambers [12, 13]. Furthermore, in some population-based studies, diabetic subjects had statistically significant higher IOP readings compared to nondiabetics [14]. Given the inverse correlation between preoperative IOP and ACD with postoperative IOP changes after cataract surgery [7–11], we hypothesized that diabetic patients could benefit from greater IOP reductions after phacoemulsification when compared to nondiabetics. However, the increased resistance to aqueous humor outflow caused by the hyperglycemia-induced overexpression of fibronectin in the trabecular meshwork could limit this hypotensive effect [15]. To the best of our knowledge, no prospective study specifically addressed the IOP-lowering effect of cataract surgery in diabetic subjects.

This study was designed to assess the IOP and AS biometric changes that occur following uneventful phacoemulsification in nonglaucomatous eyes with open-angles from nondiabetic and type 2 diabetic patients. In addition, it aimed to determine which factors may predict greater IOP reduction after surgery.

## 2. Methods

### 2.1. Participants

In this prospective observational study, type 2 diabetic patients with different stages of diabetic retinopahty (DR) and controls, aged 50 or older, were consecutively recruited from the Cataract and Refractive Surgery Unit of the Ophthalmology Department of Centro Hospitalar Universitário São João between September 2015 and March 2016. Informed consent was obtained from each participant before inclusion in the study. The study protocol was approved by the local Ethics Committee of Health and followed the tenets of the Declaration of Helsinki.

Full inclusion criteria are described elsewhere [16]. The exclusion criteria included prior eye surgery or trauma; any eye corneal, retinal or optic nerve pathology except DR; mature cataracts (brown/white) [17]; Goldmann applanation tonometry (IOP-GAT) > 25 mmHg; preoperative ACA in Scheimpflug tomography <20°; pseudoexfoliation syndrome; and current treatment with any form of steroids. Diabetic patients were excluded from the analysis if they had severe nonproliferative diabetic retinopathy (NPDR), proliferative DR (PDR), or diabetic macular edema (DME). No cases of intraoperative complications or use of adjunctive procedures (e.g., adjuvant intravitreal treatment with anti-VEGF or steroids) were included [16].

### 2.2. Sample Size Calculation

For a type I error of 0.05 and type II error of 0.20 (80% power), considering a mean difference of absolute IOP change ≥ 1.5 mmHg to be significant between the 2 groups and assuming the standard deviation (SD) for non-DM group of 2.5 mmHg, the minimal required sample size would be 44 subjects in each group [11, 17].

### 2.3. Study Protocol

#### 2.3.1. Preoperative Assessment

All patients underwent preoperative evaluation, within 2 weeks prior to cataract surgery, including general anamnesis and comprehensive ophthalmologic examination (visual acuity testing, refraction, slit-lamp examination, intraocular pressure measurement and indirect ophthalmoscopy).

For ocular biometry, the IOL Master® 500 (software version 7.7) was used. Anterior segment morphometry was evaluated using Pentacam® HR (software version 1.20r87). Measurements were repeated as necessary until high-quality images were obtained. All measurements were performed by an experienced operator (JB) under standard dim light conditions, without cyclopegia, and the patients were told to blink immediately before each examination [16].

Intraocular pressure was averaged from the two measurements performed using Goldmann applanation tonometry. If the two IOP values differed by more than 2 mmHg, then a third measurement was made and the median value was the one considered. The type of cataract (cortical, nuclear, and posterior subcapsular) and nucleus opacity grade (1 (mild) to 4 (white/brown) severity grading system) were classified after pupillary dilatation. The grade of DR was assessed in all diabetic patients using 7 standard ETDRS fundus photographs [18].

At the end of the baseline visit, an experienced nurse recorded vital signs and collected blood samples, by venous puncture, for serum HbA1c analysis [16].

#### 2.3.2. Surgical Technique

All cataract surgeries were performed under topical anesthesia by experienced surgeons. The subjects underwent standard coaxial 2.75 mm clear cornea phacoemulsification technique (Model Infiniti; Alcon Laboratories, Inc., Fort Worth, TX, USA) with in-the-bag 1-piece acrylic posterior chamber IOL (Acrysof® SA60AT (Alcon Laboratories, Inc., Fort Worth, TX, USA) or Akreos® Adapt lens (Baush & Lomb, Inc., Rochester, NY, USA)) implantation. The ophthalmic viscoelastic device used in all patients was Provisc® (sodium hyaluronate 10%; Alcon Laboratories, Inc.).

The same postoperative medication was prescribed to all the patients, and it consisted of 1 mg/ml dexamethasone, 0.3 mg/ml flurbiprofen, and 5 mg/ml levofloxacin eye drops, five times daily 1 week and then tapered gradually over 3 weeks.

#### 2.3.3. Postoperative Assessment

Patients were evaluated at 1 and 6 months postoperatively using a similar protocol to the baseline visit, with the exception of ocular biometry. Each subject was reexamined at the same time of the baseline visit.

### 2.4. Devices

#### 2.4.1. IOLMaster® 500 (Carl Zeiss Meditec, Jena, Germany)

The IOLMaster® 500 is a partial coherence interferometer used for ocular biometry. It automatically measures the anterior corneal keratometry and the axial length, which are fundamental for IOL power calculation and implantation, and have shown a high intra- and interobserver reproducibility [19].

#### 2.4.2. Pentacam® HR (Oculus, Wetzlar, Germany)

The Pentacam uses a single 180-degree rotating Scheimpflug camera and a monochromatic blue slit-light source (475 nm) combined with a static camera (for the correction of any eye movement) to generate a three-dimensional high-resolution (HR) image of the anterior segment. The software enables accurate and reproducible automatic evaluation of central corneal thickness (CCT, measured at corneal apex), ACD (from endothelium to anterior surface of lens), ACV (over a diameter of 10 mm centered on the corneal apex), and ACA (the smallest angle in the Scheimpflug images taken in the horizontal section) in phakic eyes [6].

In pseudophakic eyes, anterior IOL surface may occasionally be mistaken with the iris or the IOL-related light reflex; for that reason, postoperative ACD was manually measured from the central corneal endothelium apex to the anterior IOL surface by the same investigator (DR) after adjusting the contrast of the Scheimpflug image [8, 20]. The Scheimpflug image selected for measurement was the one that provided visualization of the whole IOL optic. The value was averaged after 3 consecutive measurements.

### 2.5. Data and Statistical Analyses

Intraoperative parameters recorded included cumulative dissipated energy (CDE), which represents the amount of ultrasound energy delivered to the eye during the surgery. To determine whether preoperative IOP had an effect on the postoperative IOP change, patients were stratified into five subgroups based on preoperative IOP: 10–14, 15-16, 17-18, 19-20, and 21–25 mmHg [21–23]. Diabetic subjects were also classified into subgroups according to DM duration (<10 and ≥10 years) and HbA1c levels (<7.0 and ≥7.0%). The predictive value of previously described indices for IOP reduction after cataract surgery was investigated: pressure to depth (PD) ratio (preoperative IOP/preoperative ACD) [7]; pressure to volume (PV) ratio (preoperative IOP/preoperative ACV); and pressure to angle (PA) ratio (preoperative IOP/preoperative ACA) [8].

Statistical analysis was performed using the SPSS statistical software (version 21.0 for Mac OS; SPSS Inc., Chicago, IL, USA). In the present study, only the scheduled eye of each patient undergoing monocular cataract surgery was used for statistical analyses. Normality was assessed using distribution plots and Kolmogorov–Smirnov tests. All comparisons between the DM and non-DM groups, as well as between pre- and postoperative periods, were performed with parametric or nonparametric tests, accordingly to the normality of data. Chi-squared or Fisher's exact tests were performed for categorical variables comparison. Linear regression analysis was performed to identify the potential demographical (age and gender), clinical (DM duration and HbA1c levels), ocular (preoperative AL, CCT, ACD, ACV, and ACA), and intraoperative (cataract grade, CDE, and IOL type) variables associated with postoperative IOP changes. Statistical significance for all the analyses was set at a *p* value less than 0.05.

STROBE guidelines were followed for manuscript elaboration [24].

## 3. Results

Forty-five diabetic patients and 44 nondiabetic controls were enrolled in the study. The DM and non-DM groups were comparable with regard to their demographic and clinical characteristics, except that HbA1c levels were higher (*p* < 0.001, Mann–Whitney test) and mean cataract grade was lower (*p*=0.032, Mann–Whitney test) in the DM group (Table 1). In the DM group, a longer duration of DM was significantly associated with higher HbA1c levels (*p*=0.008, chi^2^ test).

### 3.1. Intraocular Pressure Comparisons

Mean preoperative IOP was 17.8 ± 3.1 mmHg and 16.9 ± 2.9 mmHg in DM and non-DM groups, respectively (*p*=0.188). IOP was observed to be significantly lower than preoperative value at both 1 and 6 months of follow-up in both groups (*p* < 0.001, paired *t*-test). There were no statistically significant differences in IOP variation between groups (Table 2).

Of the 89 eyes, 73 eyes (82%) demonstrated IOP reduction (mean decrease −3.6 ± 2.1 mmHg), 5 eyes (6%) experienced no change in IOP, and 11 eyes (12%) experienced IOP increase (mean increase +2.4 ± 1.3 mmHg). The mean baseline IOP of eyes that demonstrated IOP reduction (18.1 ± 2.7 mmHg; 95% CI, 17.4–18.7 mmHg) was significantly higher than those that demonstrated IOP elevation (14.2 ± 1.5 mmHg; 95% CI, 13.1–15.2 mmHg; *p* < 0.001, independent samples *t*-test). No group differences were observed with regard to the probability of either an increased or decreased IOP 6 months after surgery (*p*=0.767, Fisher's exact test).

**Figure 1:**
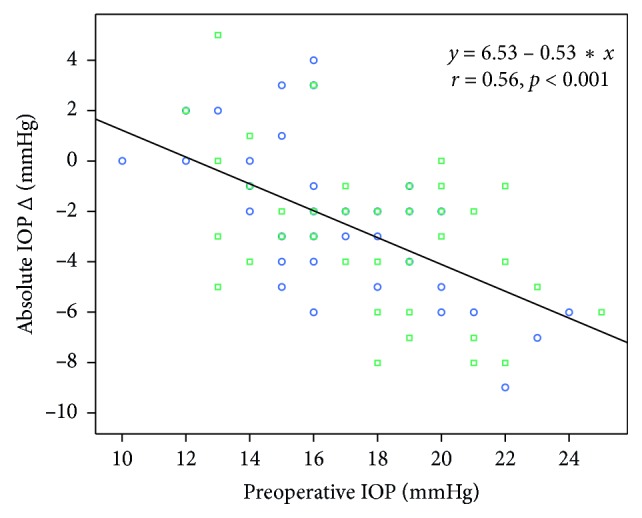
Scatterplot showing a linear relationship between preoperative IOP and absolute IOP change in both DM and non-DM groups. DM, diabetes mellitus; IOP, intraocular pressure. *x* = preoperative IOP; *y* = absolute IOP Δ; *r* = Pearson's correlation coefficient. Circles, non-DM subjects; squares, DM subjects.

A higher IOP at baseline was associated with greater IOP reduction 6 months after surgery in both DM and non-DM groups (Pearson's correlation coefficient 0.551; *p* < 0.001 vs. 0.462; *p* < 0.002, respectively) (Figure 1). The largest decrease in postoperative IOP occurred in the subgroup with the highest preoperative IOP (21–25 mmHg: −4.8 ± 2.7 in DM group (*n* = 9) and −7.0 ± 1.4 in non-DM group (*n* = 4)); while in the group with the lowest preoperative IOP (10–14 mmHg), the postoperative IOP remained essentially unchanged (−0.4 ± 3.2 in DM group (*n* = 9) and +0.1 ± 1.5 in non-DM group (*n* = 7)). There was no statistically significant difference between IOP subgroups regarding AS changes (Table 3).

There were no statistically significant differences between subgroups of DM duration or HbA1c levels in the DM subjects.

### 3.2. Scheimpflug Tomography Comparisons

#### 3.2.1. Central Corneal Thickness (CCT) Comparisons

There were no statistically significant differences between groups for the CCT measurements preoperatively, at 1- and 6-month follow-up (Table 2). The mean postoperative CCT at 1 and 6 months did not change significantly from the mean preoperative level in both DM and non-DM groups (paired *t*-test; *p* > 0.05).

#### 3.2.2. Anterior Chamber Depth (ACD) Comparisons

Mean preoperative ACD was 2.6 ± 0.4 mm and 2.7 ± 0.4 mm in DM and non-DM groups, respectively (*p*=0.135). ACD was observed to be significantly greater than preoperative value at 1 and 6 months of follow-up in both groups (*p* < 0.001, paired *t*-test). No group differences were observed with regard to ACD variations at 1 and 6 months after surgery (Table 2).

#### 3.2.3. Anterior Chamber Volume (ACV) Comparisons

Mean preoperative ACV was 126.4 ± 33.3 mm^3^ and 138.0 ± 35.4 mm^3^ in DM and non-DM groups, respectively (*p*=0.116). ACV was observed to be significantly greater than preoperative value at 1 and 6 months of follow-up in both groups (*p* < 0.001, paired *t*-test). No group differences were observed with regard to ACV variations at 1 and 6 months after surgery (Table 2).

#### 3.2.4. Anterior Chamber Angle (ACA) Comparisons

Mean preoperative ACA in DM group was significantly lower compared with non-DM group (30.2 ± 5.5° vs. 33.0 ± 5.9°, respectively (*p*=0.022)). ACA was observed to be significantly greater than preoperative value at 1 and 6 months of follow-up in both groups (*p* < 0.001, paired *t*-test), but no group differences were observed at final visit. Similarly, there were no statistical differences in ACA variations at 1 and 6 months after surgery (Table 2).

### 3.3. Factors Influencing the Postoperative IOP Change

Multivariate linear regression adjusting for age, gender, axial length, diabetes mellitus, CDE, and relevant AS Scheimpflug parameters (CCT, PD, PV, and PA) showed that only preoperative IOP was significantly associated with absolute IOP reduction 6 months after surgery. IOP was found to significantly decrease on average 0.53 mmHg for every 1 mmHg increase in preoperative IOP (*p*=0.003; Table 4).

## 4. Discussion

Given the variability of the postoperative IOP response after uneventful phacoemulsification cataract surgery with posterior chamber IOL implantation reported in the literature [1], there has been a significant effort to understand the mechanisms underlying IOP changes. The information derived from basic and clinical studies has suggested that this is a multifactorial phenomenon that includes a reduction in aqueous production [23] and an improved conventional [21, 25, 26] and uveoscleral aqueous humor outflow [27].

Results from this study showed a comparable IOP reduction 6 months after cataract surgery in nonglaucomatous eyes with open angles from nondiabetic (−2.4 ± 2.8 mmHg) and type 2 diabetic patients (−2.9 ± 2.9 mmHg). In line with previous studies assessing AS morphometry changes by Scheimpflug imaging (Table 5), all eyes from both groups experienced a significant widening of the anterior chamber depth, volume, and angle, while mean CCT did not change significantly at 1 and 6 months after cataract surgery [8, 28, 29]. It should be noted that subjects' characteristics (age and ethnic differences), Scheimpflug devices (Pentacam CES [8, 28, 29] and HR [9], EAS-1000 [2], Sirius [30, 31]), and image analysis techniques were not the same in all studies. Therefore, precaution is warranted regarding direct comparisons between the studies.

Regarding postoperative ACD assessment [8, 20], the authors confirmed that the automatic analysis provided by the Pentacam software frequently resulted in erroneous measurements due to inaccuracies in the identification of IOL's anterior surface. In the current study, similarly to Dooley et al. [8], all postoperative measurements were performed manually by one of the authors. This method has been shown to have adequate repeatability and reproducibility in pseudophakic eyes [32]. Other Scheimpflug-based studies relied on the automatic evaluation [28] or did not specify the method used [9, 29].

Several aspects of anterior segment anatomy have been found to differ between DM and non-DM patients. Previous studies [12, 13] reported that diabetic subjects had shallower anterior chambers, probably secondary to an increased lens thickness. The present study confirmed that DM subjects have smaller anterior chamber angles; however, due to the relatively small population sample, the ACD and ACV differences did not reach statistical significance. Unfortunately, in our study, none of the technologies used was able to measure lens vault or thickness, and, so the influence of these important parameters on the ACD could not be ascertained.

In some population-based studies, diabetic patients had statistically significant higher IOP-GAT readings compared to nondiabetics. This finding has been attributed to an increased corneal thickness and stiffness caused by protein cross-linking resulting from advanced glycosylated end-products [14]. Moreover, Last et al. hypothesized that an elevated corneal resistance factor measured with the Ocular Response Analyzer®, as found in DM subjects, could be accompanied by an increased stiffness of the trabecular meshwork which, in turn, would cause greater resistance to aqueous humor outflow and IOP elevation [33]. In our study, the wide standard deviations of the IOP measurements or a relatively small sample size of the study populations could explain the lack of statistical differences in IOP readings between groups.

In the current study, the authors were not able to found any statistical differences regarding IOP or AS variations at 1 and 6 months between DM and non-DM subjects. The results of our univariate and multivariate linear regression analyses, which were adjusted for potential confounders, suggested no relationship between the presence of DM and long-term postoperative IOP reduction. Interestingly, at 1-month follow-up, diabetic patients had a smaller nonstatistically significant reduction of IOP compared to the non-DM group, but this relationship was inversed at 6 months. Wang et al. [25] proposed that ultrasonic vibrations from phacoemulsification could induce stress remodeling of the trabecular meshwork and then lead to IOP reduction. It is possible that, in diabetic patients, this remodeling is delayed due to the overexpression of fibronectin induced by hyperglycemia [15].

Despite the growing recognition of the importance of preoperative IOP in postoperative IOP changes following phacoemulsification, only in 2008 Poley et al., by stratifying preoperative pressures, demonstrated that postoperative IOP reduction was proportional to preoperative IOP [21]. The present study, adopting the same methodology, allowed the authors to conclude that eyes with the highest mean preoperative IOP had the greatest magnitude of decrease and eyes with the lowest mean preoperative IOP had an insignificant mean IOP reduction or a mild IOP elevation [21–23]. Not only that, but it also showed that AS changes did not differ significantly between the subgroups, which suggest that preoperative IOP is the major factor that determines IOP reduction after phacoemulsification.

Predictive models of IOP reduction based on preoperative factors represent an important attempt to improve decision-making process of cataract surgery, in particular for ocular hypertension or glaucoma subjects with open-angles. Anterior segment-specific factors, including anterior chamber anatomy (depth [7, 8, 10, 11, 31], volume [8, 23, 31], and angle [4, 8, 23, 34]), iris (cross-sectional area and convex shape) [35], and lens factors (thickness [23], position [10, 11], and vault [5, 34]) are likely important predictors of the expected IOP change. However, the clinical significance and relationship between those variables continue to be controversial. In our study, PD, PV, and PA ratios were significantly associated with postoperative IOP change in univariate analysis; however, the effect was no longer significant after multivariate adjustment [5, 23]. In the multivariate model, the only significant predictor of postoperative IOP changes was preoperative IOP [36, 37].

Few studies have investigated the impact of phacoemulsification parameters on postoperative IOP changes. Similar to Lee et al. [17], our analysis failed to demonstrate any significant relationship between the amount of CDE and the IOP variations. A study by DeVience and colleagues [38] was able to show a significant correlation between phacoemulsification time and postoperative IOP reduction 24 months postoperatively. However, these findings were not confirmed by Pradhan et al. [35].

Limitations to this study include IOP measurement at a single visit preoperatively [37]. Also, the inclusion of cataract surgeries performed by multiple surgeons may have introduced some variability; nevertheless, no significant intersurgeon differences were observed. Another drawback is the fact that the present study excluded subjects with more advanced stages of DR (NPDR with maculopathy and PDR), mature cataracts, and complicated surgeries; therefore, we cannot make any considerations in those particular groups of patients. Finally, only Caucasian patients were included.

In conclusion, this study found that IOP reduction 6 months following uneventful phacoemulsification was strongly correlated with preoperative IOP in nonglaucomatous eyes with open-angles, without any difference between DM and non-DM groups. Additional studies may support our findings, and this topic needs further evaluation, inclusive with other AS imaging devices.

## Figures and Tables

**Table 1:** Demographic and clinical characteristics of the study population.

	DM group (*n* = 45)	Non-DM group (*n* = 44)	*p*
Age (y)	72.7 ± 5.7	70.6 ± 6.3	0.106^1^
Female (*n*)	28 (63%)	27 (61%)	0.934^3^
Right eyes (*n*)	22 (49%)	29 (66%)	0.105^3^
BMI (kg/m^2^)	28.2 ± 3.9	27.9 ± 5.2	0.763^1^
Smoking history (*n*)	10 (22%)	17 (39%)	0.092^3^
HbA1c levels (%)	6.8 ± 1.0	5.5 ± 0.4	<0.001^*∗*2^
Duration of diabetes (y)	9.1 ± 8.0	n/a	n/a
DR stage (*n*)			
No apparent DR	39 (87%)	n/a	n/a
Mild to moderate NPDR	6 (13%)		
Oral antidiabetic agents (*n*)	43 (96%)	n/a	n/a
Insulin treatment (*n*)	7 (16%)	n/a	n/a
Axial length (mm, preoperatively)	22.9 ± 0.7	23.0 ± 0.8	0.8551
Intraoperative data			
Cataract grade	1.6 ± 0.6	1.9 ± 0.6	0.032^*∗*2^
CDE	9.3 ± 7.1	9.3 ± 6.4	0.997^1^
IOL power	22.1 ± 1.6	22.2 ± 1.8	0.687^2^
Acrysof®/Akreos®	37/8	38/6	0.592^3^

Data were derived from independent samples *t*-test^1^, Mann–Whitney test^2^, and chi-square^3^ test. Continuous variables are reported as mean ± standard deviation. ^*∗*^*p* < 0.05, statistical significance. BMI, body mass index; CDE, cumulative dissipated energy; DM, diabetes mellitus; DR, diabetic retinopathy; NPDR, nonproliferative DR; IOL, intraocular lens; mm, millimeters; n/a, not applicable; y, years.

**Table 2:** Pre- and postoperative measurements in the DM and non-DM groups.

	DM group (*n* = 45)	Non-DM group (*n* = 44)	*p*
*CCT* (*μ*m)			
Preoperatively	559.4 ± 37.7	558.3 ± 29.2	0.885^1^
1 mo	562.5 ± 35.2	559.6 ± 28.5	0.671^1^
6 mo	554.1 ± 32.1	565.2 ± 31.7	0.107^1^

*IOP-GAT* (mmHg)			
Preoperatively	17.8 ± 3.1	16.9 ± 2.9	0.188^1^
Δ1 mo	−1.7 ± 2.9	−2.2 ± 2.5	0.347^1^
Δ6 mo	−2.9 ± 2.9	−2.4 ± 2.8	0.410^1^

*ACD* (mm)			
Preoperatively	2.6 ± 0.4	2.7 ± 0.4	0.135^1^
Δ1 mo	+1.3 ± 0.3	+1.3 ± 0.3	0.675^1^
Δ6 mo	+1.4 ± 0.3	+1.3 ± 0.3	0.438^1^

*ACV* (mm^3^)			
Preoperatively	126.4 ± 33.3	138.0 ± 35.4	0.116^1^
Δ1 mo	+50.1 ± 22.65	+48.2 ± 22.1	0.696^1^
Δ6 mo	+52.6 ± 23.4	+49.3 ± 23.6	0.501^1^

*ACA* (degree)			
Preoperatively	30.2 ± 5.5	33.0 ± 5.9	0.022^*∗*1^
Δ1 mo	+13.5 ± 4.9	+12.5 ± 5.6	0.356^1^
Δ6 mo	+14.2 ± 5.1	+12.8 ± 6.1	0.231^1^

Data were derived from independent samples *t*-test^1^, Mann–Whitney test^2^, and chi-squared test^3^. Continuous variables are reported as mean ± standard deviation. ^*∗*^*p* < 0.05, statistical significance. ^a^CCT measured by Pentacam at corneal vertex. ACA, anterior chamber angle; ACD, anterior chamber depth; ACV, anterior chamber volume; CCT, central corneal thickness; GAT, Goldmann applanation tonometry; K, keratometry; IOP, intraocular pressure; mo, month; PD, pressure-to-depth ratio; Δ, variation.

**Table 3:** Subgroup analysis of DM and non-DM groups according preoperative IOP subgroups.

	Pre-op. IOP	Eyes (*n*, %)	Age (y)	IOP pre-op. (mmHg)	IOP Δ 6 mo (mmHg)	ACD pre-op (mm)		ACD Δ 6 mo (mm)	ACV pre-op (mm^3^)	ACV Δ 6 mo (mm^3^)	ACA pre-op (°)	ACA Δ 6 mo (°)
DM group (*n* = 45)	21–25	9 (20%)	73 ± 7	22.0 ± 1.3	−4.8 ± 2.7	2.5 ± 0.4	+1.5 ± 0.2		129 ± 41	+59 ± 13	30 ± 5	+15 ± 7
	19-20	11 (24%)	72 ± 4	19.4 ± 0.5	−3.3 ± 2.3	2.5 ± 0.3	+1.2 ± 0.4		118 ± 22	+46 ± 21	29 ± 5	+13 ± 5
	17-18	9 (20%)	70 ± 7	17.7 ± 0.5	−4.1 ± 2.3	2.5 ± 0.3	+1.4 ± 0.2		119 ± 33	+55 ± 21	29 ± 4	+15 ± 5
	15-16	7 (16%)	72 ± 4	15.6 ± 0.5	−1.7 ± 2.1	2.5 ± 0.4	+1.5 ± 0.3		122 ± 35	+59 ± 27	27 ± 5	+16 ± 3
	10–14	9 (20%)	77 ± 4	13.3 ± 0.7	−0.4 ± 3.2	2.8 ± 0.3	+1.3 ± 0.2		146 ± 35	+46 ± 29	35 ± 7	+12 ± 5
	*p*	0.676^1^	0.140^2^	<0.001^*∗*2^	0.028^*∗*2^	0.320^2^	0.109^2^		0.401^2^	0.694^2^	0.088^2^	0.416^2^

Non-DM group (*n* = 44)	21–25	4 (9%)	76 ± 12	22.5 ± 1.3	−7.0 ± 1.4	2.6 ± 0.4	+1.3 ± 0.2		116 ± 23	+50 ± 27	29 ± 4	+9 ± 5
	19-20	8 (18%)	70 ± 5	19.5 ± 0.5	−3.4 ± 2.1	2.7 ± 0.5	+1.3 ± 0.3		145 ± 41	+39 ± 31	35 ± 6	+11 ± 8
	17-18	10 (23%)	70 ± 6	17.6 ± 0.5	−2.7 ± 1.0	2.7 ± 0.3	+1.3 ± 0.3		139 ± 24	+49 ± 16	35 ± 5	+12 ± 5
	15-16	15 (34%)	70 ± 6	15.5 ± 0.5	−1.8 ± 3.1	2.6 ± 0.4	+1.4 ± 0.3		135 ± 35	+53 ± 22	31 ± 6	+15 ± 5
	10–14	7 (16%)	71 ± 4	12.7 ± 1.5	+0.1 ± 1.5	2.8 ± 0.5	+1.3 ± 0.4		152 ± 48	+52 ± 29	34 ± 6	+14 ± 7
	*p*	0.759^1^	0.928^2^	<0.001^*∗*2^	0.001^*∗*2^	0.797^2^	0.763^2^		0.469^2^	0.715^2^	0.143^2^	0.516^2^

Data were derived from Fisher's exact test^1^ and Kruskal–Wallis test^2^. Continuous variables are reported as mean ± standard deviation. ^*∗*^*p* < 0.05, statistical significance. ACA, anterior chamber angle; ACD, anterior chamber depth; ACV, anterior chamber volume; IOP, intraocular pressure; mm, millimeters; mo, months; Δ, variation.

**Table 4:** Uni- and multivariate regression analyses of the relative effects of the baseline variables on postoperative IOP change.

Parameter	Absolute IOP Δ (mmHg)			
	Univariate		Multivariate	
	B (95% CI)	*p*	B (95% CI)	*p*
Age (y)	−0.003 (−0.10 to +0.10)	0.960	−0.16 (−0.11 to +0.08)	0.747
Gender (female)	+0.84 (−0.39 to 2.07)	0.177	+0.89 (−0.34 to +2.12)	0.155
DM	−0.50 (−1.71 to +0.70)	0.410	−0.059 (−1.14 to +1.02)	0.914
Axial length (mm)	+0.06 (−0.75 to +0.87)	0.888	−0.12 (−1.07 to +0.82)	0.795
Pre-op CCT (*μ*m)	−0.02 (−0.03 to +0.003)	0.102	−0.01 (−0.02 to +0.01)	0.589
Pre-op IOP (mmHg)	−0.53 (−0.70 to −0.36)	<0.001^*∗*^	−0.53 (−0.88 to −0.19)	0.003^*∗*^
PD ratio	−0.72 (−1.03 to −0.40)	<0.001^*∗*^	−0.02 (−1.51 to +1.46)	0.976
PV ratio	−17.72 (−28.29 to −7.14)	0.001^*∗*^	−8.02 (−42.09 to +26.04)	0.640
PA ratio	−6.41 (−9.87 to −2.95)	<0.001^*∗*^	+2.64 (−4.69 to +9.96)	0.476

Data were derived from linear regression models. Continuous variables are reported as mean ± standard deviation. ^*∗*^*p* < 0.05, statistical significance. CCT, central corneal thickness; DM, diabetes mellitus; IOP, intraocular pressure; PA, pressure to angle ratio; PD, pressure to depth ratio; PV, pressure to volume ratio; mm, millimeters; y, years. The remaining variables (DM duration, HbA1c levels, CDE, cataract grade, and IOL type) did not influence the model and were excluded.

**Table 5:** Review of studies assessing the IOP reduction and AS changes after phacoemulsification by Scheimpflug imaging.

Study (year)	Patients (eyes)	Glaucoma	Age (y)	Female (*n*)	Follow-up	IOP pre-op (mmHg)	IOP Δ (mmHg)	ACD pre-op (mm)	ACD Δ (mm)	ACV pre-op (mm^3^)	ACV Δ (mm^3^)	ACA pre-op (°)	ACA Δ (°)
Hayashi et al. [2] (2000)^a^	77 (77)	ACG	74 ± 8	56 (73%)	12 mo	21.4 ± 3.9	−6.1 ± 3.9^*∗*^	1.9 ± 0.3	+2.1 ± 0.4^*∗*^	n.r.	n.r.	19 ± 4	+18 ± 5^*∗*^
	73 (73)	OAG	74 ± 7	39 (53%)		20.5 ± 5.4	−4.4 ± 4.3^*∗*^	2.8 ± 0.4	+1.5 ± 0.4^*∗*^			28 ± 5	+10 ± 7^*∗*^
	74 (74)	No	72 ± 11	40 (54%)		17.3 ± 3.3	−1.0 ± 4.1^*∗*^	2.9 ± 0.4	+1.4 ± 0.6^*∗*^			27 ± 6	+11 ± 8^*∗*^

Uçakhan et al. [28] (2009)^b^	44 (44)	No	66 ± 8	n.r.	3 mo	15.8 ± 3.7	−2.6 ± n.r.^*∗*^	3.0 ± 0.8	+0.9 ± n.r.^*∗*^	165 ± 50	+36 ± n.r.^*∗*^	36 ± 10	+6 ± n.r.^*∗*^

Doganay et al. [29] (2010)^b^	34 (42)	No	65 ± 8	8 (24%)	6 mo	14.6 ± 2.5	−2.8 ± n.r.^*∗*^	2.8 ± 0.4	+1.9 ± n.r.^*∗*^	145 ± 44	+46 ± n.r.^*∗*^	33 ± 6	+10 ± n.r.^*∗*^
Dooley et al. [8] (2010)^b^	101 (101)	No	69 ± 11	62 (62%)	6 weeks	14.8 ± 3.1	−2.5 ± 3.2^*∗*^	2.7 ± 0.4	+1.1 ± 0.5 ^*∗*^	142 ± 49	+54 ± 27^*∗*^	30 ± 6	+13 ± 7 ^*∗*^

Mota et al. [9] (2011)^c^	30 (31)	No	73 ± 8	18 (58%)	1 mo	20.5 ± 4.4^e^	−3.9 ± 5.6^*∗*^	2.8 ± 0.5	+1.5 ± 0.7 ^*∗*^	n.r.	n.r.	32 ± 6	+13 ± 6 ^*∗*^

Takmaz et al. [30] (2012)^d^	54 (56)	No	66 ± 10	30 (56%)	1 mo	14.6 ± 3.5	−4.2 ± n.r.^*∗*^	2.7 ± 0.4	+0.8 ± n.r.^*∗*^	144 ± 49	+49 ± n.r.^*∗*^	42 ± 8	+11 ± n.r.^*∗*^

Şimşek et al. [31] (2016)^d^	132 (132)	No	64 ± 13	46 (35%)	3 mo	14.7 ± 2.6	−2.4 ± n.r.^*∗*^	2.8 ± 0.4	+0.7 ± n.r.^*∗*^	125 ± 26	+38 ± n.r.^*∗*^	42 ± 7	+9 ± n.r.^*∗*^

Statistically significant difference at *p* < 0.05. AS, anterior segment; CCT, central corneal thickness; n.r., not reported; SD, standard deviation; SE, standard error; US, ultrasound pachymetry; y, years; mmHg, millimeters of mercury. ^a^AS evaluation with EAS-1000 (Scheimpflug videophotography system); ^b^AS evaluation with Pentacam CES; ^c^AS evaluation with Pentacam HR; ^d^AS evaluation with Sirius; ^e^IOP was measured with ocular response analyzer.

## Data Availability

The data used to support the findings of this study are available from the corresponding author upon request.
